# Youth Study Recruitment Using Paid Advertising on Instagram, Snapchat, and Facebook: Cross-Sectional Survey Study

**DOI:** 10.2196/14080

**Published:** 2019-10-09

**Authors:** Kelsey Lynett Ford, Tashuna Albritton, Tara A Dunn, Kacy Crawford, Jessica Neuwirth, Sheana Bull

**Affiliations:** 1 The mHealth Impact Lab Colorado School of Public Health Aurora, CO United States; 2 Anschutz Medical Campus University of Colorado Aurora, CO United States; 3 School of Medicine The City College of New York New York, NY United States; 4 Colorado Department of Public Health & Environment Denver, CO United States

**Keywords:** social media, youth, surveys and questionnaires

## Abstract

**Background:**

The use of paid social media advertising for targeted study recruitment is an effective strategy in health research and evaluation, specifically to reach diverse youth participants. Although the literature adequately describes the utility of Facebook in recruitment, limited information exists for social media platforms that are more popular with youth, specifically Instagram and Snapchat.

**Objective:**

This paper outlines a paid advertising approach using Instagram, Snapchat, and Facebook to evaluate a statewide youth marijuana prevention campaign. The objective of this study was to compare recruitment metrics across Instagram, Snapchat, and Facebook for two surveys documenting youth knowledge, attitudes, and behaviors related to retail marijuana in Colorado post legalization. In addition, the study assessed the feasibility of using Instagram and Snapchat as effective additions to Facebook for youth study recruitment.

**Methods:**

A social media recruitment strategy was used to conduct two cross-sectional surveys of youth, aged 13 to 20 years, in Colorado. Geographically targeted ads across 3 social media platforms encouraged the completion of a Web-based self-administered survey. Ad Words and Snap Ads were used to deploy and manage advertising campaigns, including ad design, placement, and analysis. Ad costs and recruitment metrics (ie, impressions, link clicks, and conversion rates) were calculated across the three social media platforms.

**Results:**

Over two 1-month periods, 763,613 youth were reached (ie, impressions), 6089 of them clicked survey links (ie, clicks), and 828 eligible youth completed surveys about knowledge, attitudes, and behaviors related to retail marijuana. Instagram converted 36.13% (803/2222) of impressions to clicks (ie, conversion rate) in the first survey and 0.87% (864/98982) in the second survey. Snapchat generated the most impressions and link clicks, but it did so with the lowest conversion rate for both surveys, with a 1.40% (1600/114,200) conversion rate in the first survey and a 0.36% (1818/504700) conversion rate in the second survey. Facebook maintained a consistent conversion rate of roughly 2% across both surveys, despite reductions in budget for the second survey. The cost-per-click ranged between US $0.25 and $0.37 across the three platforms, with Snapchat as both the most cost-effective platform in the first survey and the most expensive platform in the second survey.

**Conclusions:**

Recruitment and enrollment outcomes indicate the use of Instagram and Snapchat, in addition to Facebook, may be a modern, useful, and cost-effective approach to reach youth with surveys on sensitive health topics. As the use of Facebook declines among youth, the use of more popular social media platforms can augment study recruitment for health research and evaluation efforts.

## Introduction

In the United States, social media is becoming increasingly valuable to recruit youth participants in health research and program evaluation. Evidence supports that social media is an advantageous approach to recruit hard-to-reach populations and individuals with specific disease states [1-6]. Some studies find social media recruitment strategies more cost effective, compared with traditional enrollment methods [2]. Many reviews suggest that using these platforms for study recruitment is effective in reaching adolescents and young adults [3,7,8]. These reviews demonstrate that youth are more forthcoming with self-administered surveys, using technology platforms, particularly when it comes to disclosing information on sensitive topics [9]. 

The universal use of social media among younger populations motivates researchers to utilize Web-based strategies. According to the US Department of Health and Human Services and the PEW Research Center, 71% of teens use more than one social media platform; finding Facebook is no longer the social media platform of choice for young people [6,10]. In 2018, the social media landscape shifted, reporting YouTube (85%), Instagram (72%), and Snapchat (69%) as the most utilized social media platforms by young people [6,11]. As social media preferences evolve, a continued understanding of how to reach youth is critical to eliciting information on health behavior.

Targeted paid advertising on social media platforms is a useful way to increase the reach and diversity of young study participants. Existing literature describes the utility of Facebook in youth recruitment [1,2,10,12-16], but there is limited understanding about the role of other (more popular) social media platforms, including Instagram and Snapchat. This paper outlines a paid advertising approach using Instagram, Snapchat, and Facebook to reach and enroll 2 cross-sectional samples of youth potentially exposed to a statewide marijuana prevention campaign. The objective of this study was to compare recruitment metrics (ie, impressions, link clicks, conversions, and recruitment cost per survey) across Instagram, Snapchat, and Facebook for surveys documenting youth knowledge, attitudes, and behaviors related to retail marijuana in Colorado. In addition, this study assessed the feasibility of using Instagram and Snapchat, in addition to Facebook, for youth study recruitment.

## Methods

### Overview

From December 9 to December 29, 2017, and from May 4 to June 1, 2018, the evaluation team used a social media recruitment strategy to obtain 2 cross-sectional samples of youth in Colorado. The strategy utilized paid, geographically targeted ads on Instagram, Snapchat, and Facebook to encourage the completion of a self-administered, Web-based survey. Ad images contained virtual links that prompted youth to complete an anonymous survey using Qualtrics software (Qualtrics, Provo, UT), hosted by The University of Colorado [17]. A total of 8 US $50 gift cards (ie, Target, Amazon, Spotify, and VISA) were raffled weekly to incentivize survey completion. The study was classified as program evaluation and was considered exempt from institutional review board approval; all methods adhered to ethical human subjects’ research protections.

### Eligibility Criteria

Eligibility criteria included youth (1) aged 13 to 20 years, (2) currently living in Colorado, and (3) who completed a Web-based survey.

### Ad Design

Ads for Instagram and Facebook were maintained using Ads Manager, a Web-based ad campaign creation and management tool [18]. Snapchat ads were developed and monitored using Snap Ads [19]. Each social media platform required specifications on ad delivery, ad content, design language, targeted audience, and dates of deployment (Table 1). Social media advertisement designs delineated by modality are available in Multimedia Appendix 1.

Instagram and Facebook ads utilized carousel images, headings, caption text, and hyperlinks to promote link clicks to enroll in the Web-based survey. Youth advisors from stakeholder groups provided feedback to the images, headings, and hashtags to ensure relevant and engaging content for the target population. Similarly, using a Snap Ads design template, the ad comprised a headline, animated images, and a call to action (ie, swipe) to promote participation.

Each social media platform reviewed ads before deployment. Ads underwent 3 to 5 days’ worth of appeals and iterations to meet each social media platform’s policies [18,19]. Snap Ads rejected any ads that included *Marijuana* or *Weed*. In addition, Ads Manager required multiple appeals to ensure the ads were not promoting illegal substances. The evaluation team addressed the concerns by describing the intent of the ads and removing sensitive language (ie, “weed” or “marijuana”).

**Table 1:** Social media recruitment ad summary for cross-sectional Web-based surveys.

Platform	Dates	Image	Headline	Subheading	Text	Target audience	Target location
Snapchat	12/14-12/23; 05/09-05/18; 05/29-06/01	Animated image	Be Blunt	Colorado School of Public Health	“Share your thoughts on substance use for a chance to win $50.”; “Participate anonymously”	Youth, 13-20 years old; Gender: All	Colorado, United States
Instagram	12/09-12/28; 05/04-05/29	Images (2)	Winner gets $50; Jump into the Discussion	None	“Be blunt: give us your thoughts on marijuana for a chance to #win a $50 gift card. Click here to participate anonymously. #colorado #teen #poll #survey.”	Youth, 13-20 years old; Gender: All	Colorado, United States
Facebook	12/09-12/28; 05/04-05/29	Image carousel (2)	Marijuana and Teens	Click to take an anonymous survey	“Give us your word on weed for the chance to #win a $50 gift card. Click here to take an anonymous survey now. #colorado”	Youth, 13-20 years old, from select counties; Gender: All	Alamosa (+30 miles), Colorado Springs (+30 miles), Denver (+30 miles), Fort Morgan (+30 miles), Grand Junction (+30 miles), Greeley (+30 miles), Pueblo (+30 miles), South Fork (+30 miles), Sterling (+30 miles), Vail Rd, Vail (+30 miles); Colorado, United States

### Ad Placement

Ads Manager and Snap Ads defined ad placement using ad sets. Ad sets determine the reach of the ads, specifically the location, age group, genders, and budget of the recruitment ad campaign. Ads ran during specified date ranges, targeting youth (aged 13-20 years) in Colorado (Table 1). To narrow the scope of the Facebook campaign, specific counties were targeted using a 30-mile radius for harder-to-reach rural communities.

On Facebook, ads were displayed as News Feed ads (ie, ads embedded in the dynamic news field central column) and right column ads (ie, displayed in the static column on the right side of the screen). Youth accessing Facebook on their desktop computers viewed both ads, whereas mobile users saw News Feed ads only. On Instagram, images were displayed in a linear format, labeled as a sponsored ad within the user’s personal Instagram feed. On Snapchat, images were displayed using the Stories feature; links were introduced to end users, when browsing local stories, and the survey was accessed by *swiping up*.

### Social Media Ad Costs

Advertising costs differed among social media platforms on the basis of predetermined budgets and payment methods. The evaluation team allocated lifetime and daily budgets per ad to set maximum dollar amounts spent, also referred to as bids. Purchased through an auction basis, bids charge was based on link clicks (ie, pay per click), impressions, or actions during the advertising window. Advertisers compete for ad placements using a bidding process. Higher bid amounts improve the campaigns’ chances of securing more impressions. Snap Ads and Ads Manager monitored these transactions with their respective Web-based dashboards to improve ad delivery efficiency and optimize campaign delivery [20].

Each cross-sectional survey maintained a total budget of US $1000. The evaluation team delineated daily and lifetime budgets throughout the campaign: Snapchat (US $50/day; US $300/lifetime), Instagram (US $13/day; US $350/lifetime), and Facebook (US $13/day; US $350/lifetime). During cross-sectional survey #2, a lack of impressions in Facebook ads allowed the team to reallocate dollars to Snapchat’s lifetime budget, a higher impression-generating platform, to maximize response rate. This adjustment increased Snapchat’s lifetime budget to US $670 and decreased Facebook’s lifetime budget to US $25. Excluding incentive budgets, the cost per completed survey (across all social media platforms) was US $1.62 for initial recruitment periods, and for the subsequent recruitment periods the cost per respondent was US $4.76. 

### Analysis

The dashboards for Ads Manager and Snap Ads presented recruitment outcomes for analysis. Measures included the following: (1) *impressions*, which describe the number of which ads were displayed, as indicated by the ad set target population, and this included whether the ad was clicked or not; (2) *link clicks*, the number of participant clicks to the ads’ desired destination (ie, Qualtrics survey); (3) *conversion rates*, which indicate the proportion of people exposed to the image (ie, impressions) who clicked on it (ie, link clicks); (4) standard *response rate* formulas, which calculate the screening, refusal, and completion rates for the survey based on eligible participants; (5) *recruitment cost-per-survey*, which is calculated by dividing ad costs by the total number of completed surveys.

The analytic sample excluded youth who indicated their age was younger than 13 or older than 20, and the sample excluded those who did not provide a valid Colorado zip code. To ensure participant veracity and uniqueness, the team conducted consistency checks (ie, asking age at a point in the survey and month and year of birth at another) and reviewed the internet protocol (IP) address for all participants. When the team found inconsistencies in reported age and duplicate IP addresses, the analysts removed participants from the sample. In addition, the final analytic sample removed all partially completed surveys. The team compiled and cleaned the exported data from Ads Manager, Snap Ads, and Qualtrics. Recruitment measures were calculated by ad delivery dates. Completion rates were determined on the basis of the number of eligible surveys completed via Qualtrics.

## Results

**Figure 1:**
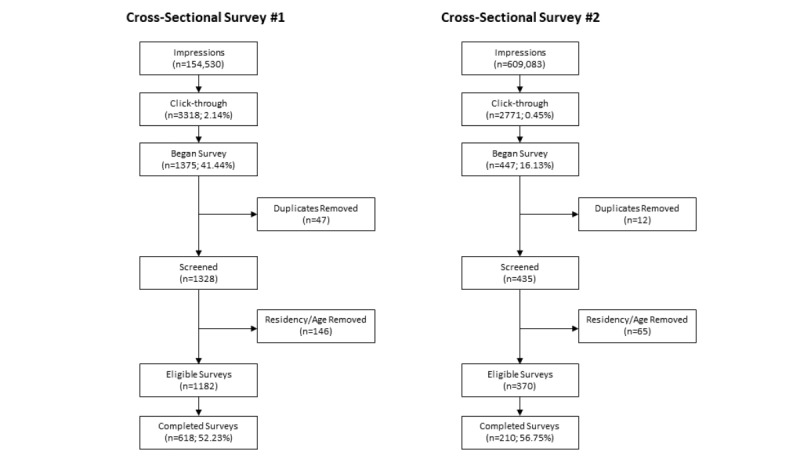
Recruitment eligibility and screening process results.

For cross-sectional survey #1, 618 participants were retained as eligible on the basis of age, residency, and survey completion (Figure 1). This represents a 52.28% (618/1182) response rate. For cross-sectional survey #2, the screening process retained 210 participants, representing a 57.8% (210/370) survey response rate.

In both surveys, older youth aged 17 to 20 years represented over half of the sample and illustrated similar racial and ethnic demographics within the region [21]. For cross-sectional survey #1, among eligible respondents (n=618), 274 (44.3%, 274/618) identified as being 13 to 16 years in age, and 344 (55.7%, 344/618) as being 17 to 20 years in age. Respondents identified as male (53.5%) or female (43.5%). Respondents identified as Hispanic/Latino (16.2%), American Indian/Native American (4.5%), Native Hawaiian/Other Pacific Islander (0.8%), Asian (2.3%), white (86.6%), and black/African American (1.9%). The majority of the sample identified as heterosexual (75.4%). Among eligible respondents in the second cross-sectional survey (n=210), 91 (43.3%, 91/210) identified as being 13 to 16 years in age, and 119 (56.7%, 119/210) as being 17 to 20 years in age. Primarily, respondents identified as male (52.9%) or female (42.4%). Respondents identified as Hispanic/Latino 39 (18.6%), American Indian/Native American 3.8%), Native Hawaiian/Other Pacific Islander (4.3%), Asian (4.3%), white (87.1%), and black/African American (3.8%). The majority of the sample identified as heterosexual (69.5%).

 Recruitment metrics for both cross-sectional surveys included impressions, link clicks, conversion rates, advertising costs, and costs per link click (Tables 2 and 3). For both data collection periods, Snapchat generated the most impressions and link clicks among the social media platforms.

**Table 2:** Summary of social media recruitment metrics (cross-sectional survey #1).

Modality	Dates	Impressions	Link clicks	Conversion rate (%)	Ad costs (US $)	Cost per link click (US $)
Instagram	12/09-12/28	2222	803	36.13	267.26	0.33
Snapchat	12/14-12/23	114,200	1600	1.40	400.00	0.25
Facebook	12/09-12/29	38,108	915	2.40	274.56	0.30
Total	—^a^	154,530	3318	—	941.82	0.28

^a^Data not applicable.

**Table 3:** Summary of social media recruitment metrics (cross-sectional survey #2).

Modality	Dates	Impressions	Link clicks	Conversion rate (%)	Ad costs (US $)	Cost per link click (US $)
Instagram	05/04-05/29	98,982	864	0.87	300.00	0.34
Snapchat	05/09-05/18; 05/29-06/01	504,700	1818	0.36	674.00	0.37
Facebook	05/04-05/29	5401	89	1.64	25.44	0.28
Total	—^a^	609,083	2771	—	999.44	0.36

^a^Data not applicable.

In cross-sectional survey #1, Snapchat and Facebook had a higher number of impressions and link clicks than Instagram; however, Instagram outperformed Snapchat and Facebook in conversion rates. Instagram’s high conversion rate (ie, 36%) remained an outlier. In cross-sectional survey #2, Instagram and Snapchat had a higher number of impressions and link clicks than Facebook. Although Instagram and Snapchat conversion rates were lower in the second survey, Facebook conversion rate was consistent in both the first and second survey. It is important to note that for the second survey, the team decreased the budget spending for Facebook ads and increased the budget for Instagram and Snapchat ads. Response rates were lower in subsequent recruitment periods. Instagram and Snapchat had a marked increase in the number of impressions and a moderate increase in number of link clicks in the second survey. Although Snapchat impressions were the highest among the social media platforms, costs per link click were the most expensive for cross-sectional survey #2.

## Discussion

### Principal Findings

This paper outlined a paid advertising recruitment strategy, comparing recruitment across Instagram, Snapchat, and Facebook for surveys documenting youth knowledge, attitudes, and behaviors related to retail marijuana in Colorado. Although retail marijuana is legalized in Colorado, it remains illegal for those under 21. Obtaining a mechanism to engage with youth and document marijuana knowledge, attitudes, and behavior is critical, particularly where retail marijuana use is legal for older populations [22]. Social media platforms are useful mechanisms to reach youth and understand their illicit behaviors, given broad reach and the opportunity to share information anonymously [23]. As youth move away from older social media platforms and adopt the use of newer versions on the Web, additional research is needed to determine if Web-based recruitment strategies are equally effective across diverse social media platforms. In health-related studies that have incorporated social media platforms (ie, MySpace, Facebook, Instagram, and Twitter) for recruitment, Facebook proves the most successful platform, compared with MySpace, Instagram, and Twitter, across age groups [5,14]. Both Instagram and Snapchat are the more recent social media platforms that should also be further examined for recruitment capabilities.

Thus, this study expanded on current evidence-based social media recruitment practices and included social media platforms that are currently more popular and relevant to youth (ie, Instagram and Snapchat) [5,24-26]. Differences were observed across platforms in youth recruitment in the 2 Web-based cross-sectional surveys; demonstrating that higher than typical numbers of youth who were exposed to our Web-based ads clicked on them [27]. The second survey had a high response rate for Web-based survey research, which typically ranges between 10% and 15% [28], but it ultimately yielded a lower response rate compared with the first survey [28].

The Ads Manager dashboard displayed few impressions on Facebook, which the evaluation team inferred was a reflection of declining interest in Facebook ads; however, Facebook still presented a feasible way to reach some younger adults (ie, 18-20 years old) for each survey. Such feasibility has been shown with a similar age groups (ie, 18-24 years old) [5]. It is possible that the reduced budget for Facebook ads for the second survey may have contributed to a lower number of impressions and link clicks. Subpopulations or hard-to-reach populations may require a larger Facebook ad budget and more time for ads to run to get a higher frequency yield [5]. In addition, it could be inferred that ads in regional counties, with high participation in the first survey, deterred participation in the second. Youth may have ignored an ad after having seen it for a previous survey. It is also possible that Facebook ads were ignored for the second survey if youth had already seen the ads in their Instagram and Snapchat feeds before they saw it on Facebook.

Instagram and Snapchat had a marked increase in the number of impressions and a moderate increase in number of link clicks in the second survey. This could have resulted from the increased ads budget, which increased ad visibility across the 2 platforms. Although a specific cost-effectiveness assessment is beyond the scope of this paper, the cost-per-survey comparison was generally consistent with what is observed in other studies [2].

Other Web-based health-related campaigns [5,23,24,29,30] demonstrated similar success in recruiting youth and young adults through Instagram and Facebook [13,22,31], although most cross-sectional studies used Facebook for recruitment [32]. In the first cross-sectional survey, the social media ads ran for 20 days and showed a total of 154,530 impressions and 3318 link clicks, across Instagram, Snapchat, and Facebook, and 618 completed surveys. In the second cross-sectional survey, social media ads ran for 28 days and showed a total of 609,083 impressions and 2771 link clicks, across the same 3 platforms, and 210 completed surveys. A study using Facebook ads only for 48 days produced a total of 144,635 impressions, 2129 link clicks, and 26 completed surveys among an adult multiethnic population [29]. A study using Facebook and Instagram ads for 1 week to recruit and reach young adults at high risk for smoking reached 324,959 individual users and resulted in 7249 link clicks, 6661 screener completions, and 1709/3357 (50.90%, eligible) completed surveys [5]. These findings suggest that recruitment and reach through a single social media platform might not yield targeted enrollment and ads using multiple platforms may be more advantageous. Other studies using social media platforms for recruitment of younger populations have instituted the use of multiple platforms (ie, Facebook, Instagram, and Twitter) [5,25] to reach target enrollment. Some studies have combined different recruitment methods to include social media, interceptive (face to face), and postal recruitment [5,31]. Though similar studies have also paid cost per click, this type of recruitment is shown to be cost effective than traditional methods [5,13,31].

This study showed the feasibility of incorporating Instagram and Snapchat to a traditional Facebook paid advertising recruitment strategy. Both Instagram and Snapchat required similar elements of ad content, design, placement, and budget considerations. Instagram utilized the same Web-based platform as Facebook (ie, Ads Manager) and streamlined logistics associated with setting up and monitoring the 2 campaigns. Not only is Instagram feasible for recruitment but it also has been associated with youth retention in a Web-based mental health and substance use interventions [26]. Thus, the initial draw to a study through social media may maintain interest in completing Web-based interventions and surveys. Although Snapchat ads required artistic animation, Snap Ads’ design templates offered user-friendly ways to create ads even for researchers lacking graphic design skills. Both Instagram and Snapchat followed similar advertising policies, which aligned with Facebook. This allowed researchers to prepare for the approval and appeal process accordingly. Finally, the differences in advertising costs across platforms were negligible. Although more robust comparisons of recruitment strategies should be investigated, findings suggest incorporating Instagram and Snapchat as an accessible and practical addition to recruiting youth on the Web for health studies.

### Limitations

Study limitations exist despite successful recruitment using social media ads. The recruitment evaluation design lacked a comparison recruitment process using in-person recruitment methods. In addition, the cross-sectional surveys gathered convenience samples; therefore, findings are not generalizable to the population of 13- to 20-year-old Coloradans. Understanding knowledge, attitudes, and behaviors of youth younger than 13 years old is critical for preventing retail marijuana intentions and use. However, because of social media advertising policies, sampling youth younger than 13 years old was not permitted. The findings only illustrate descriptive results related to social media recruitment methods for youth aged 13 to 20 years. Altering the budget in the second cross-sectional survey created a potential bias for Snapchat and Instagram success.

Although it is an unsupported hypothesis, the research team considered how external factors (eg, school holidays, final exams, and weather) may have contributed to responses across the data collection period. Such contextual factors should be considered in future social media recruitment approaches. In addition, all ads used the same images and content. Individuals who completed the survey before might have thought they could not complete it a second time. In addition, because of the anonymous survey link used in ads, there is limited understanding of which social media platform yielded the most completed surveys and which were most cost-effective. However, it would seem that the approach is more economically viable than hiring staff to recruit youth, travelling to specific recruitment locations, and spending time administering in-person surveys.

There are few methods in the scientific literature using popular social media platforms for youth recruitment, such as Instagram and Snapchat [25,33]. Although this study explored the use of modern social media platforms to reach young people, additional research is needed as technology and internet use trends continue to change.

### Strengths

This study highlighted several strengths to the health sciences literature. First, limited scholarship describes the use of Instagram and Snapchat paid advertising for youth study recruitment. This offered a significant contribution to understanding how to utilize diverse social media platforms for health-related research and evaluation. The study demonstrates the usefulness of social media recruitment in health-related research, particularly in its ability to reduce data collection time and provide rapid results about emerging public health problems, such as illegal marijuana use in states where retail marijuana sell is legal. Traditional recruitment methods may take months, thereby adding to the time it takes to collect data and disseminate results. Innovative use of Snapchat as an avenue for recruitment showed high impressions, suggesting a noteworthy method to reach young people. Second, this evaluation offered unique contributions on how social media campaigns can use multiple platforms to maximize recruitment, reach, and engagement. Third, the study contributed to the literature by describing low-cost approaches for reaching young people using paid social media advertising [5,29,31].

### Conclusions

Social media platforms can play a significant role in reaching young people for research and evaluation of youth-focused programs. These platforms are appealing to younger populations, allowing for easier design and tailoring to recruit specific populations [5,34]. The findings represent a feasible and modern approach to recruit cross-sectional samples using social media platforms beyond Facebook. A social media recruitment strategy that includes platforms most used by youth (eg, Instagram and Snapchat) can enhance Facebook recruitment approaches. Although no social media platform is a solution to study recruitment, diversifying recruitment across multiple platforms may increase response rates and improve researchers’ ability to reach youth in an efficient manner. The use of multiple platforms may also broaden the reach for subpopulations and hard-to-reach youth populations [31] and increase sample representativeness [25]. As the use of Facebook declines among youth, alternative, more popular social media platforms, such as Instagram and Snapchat, provide promise for health research and evaluation recruitment practices.

## Appendix

Multimedia Appendix 1 Social media advertisement designs delineated by modality.

## Abbreviations

IP: internet protocol
