# Supplementary material for: Youth Study Recruitment Using Paid Advertising on Instagram, Snapchat, and Facebook: Cross-Sectional Survey Study
Source: JMIR Public Health Surveill. 2019 Oct 9;5(4):e14080. doi: 10.2196/14080 (PMC6811770; doi:10.2196/14080)
Supplement: Multimedia Appendix 1 [file publichealth_v5i4e14080_app1.pdf]

Multimedia Appendices 1

Recruitment Social Media Advertisement Designs					
**Modality**	**Run Dates**	**Image/Video**	**Headline**	**Sub Heading**	**Text**
Snapchat	12/14/17-12/23/17<br><br>05/09/18-05/18/18<br><br>05/29/18-06/01/18	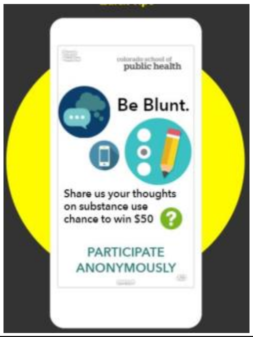	Be Blunt	Colorado School of Public Health	"Share your thoughts on substance use for a chance to win $50; participate anonymously"
Instagram	12/09-12/28<br>05/04-05/29	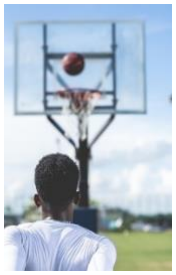	Winner gets $50	None	"Be blunt: give us your thoughts on marijuana for a chance to #win a $50 gift card. Click here to participate anonymously. #colorado #teen #poll #survey."
Instagram	12/09-12/28<br>05/04-05/29	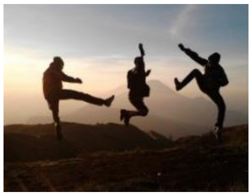	Jump into the Discussion	None	"Be blunt: give us your thoughts on marijuana for a chance to #win a $50 gift card. Click here to participate anonymously. #colorado #teen #poll #survey."
Facebook	12/08-12/29<br>05/04-05/29	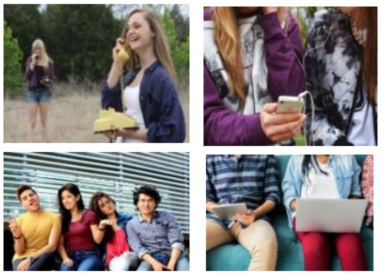	Marijuana and Teens	Click to take an anonymous survey	"Give us your word on weed for the chance to #win a $50 gift card. Click here to take an anonymous survey now. #Colorado #giveaway #poll "
